# Supplementary material for: Natural Product Library Screens Identify Sanguinarine Chloride as a Potent Inhibitor of Telomerase Expression and Activity
Source: Cells. 2022 Apr 28;11(9):1485. doi: 10.3390/cells11091485 (PMC9104802; doi:10.3390/cells11091485)
Supplement: Supplementary file 1 [file cells-11-01485-s001.zip › cells-1665800-supplementary.pdf]

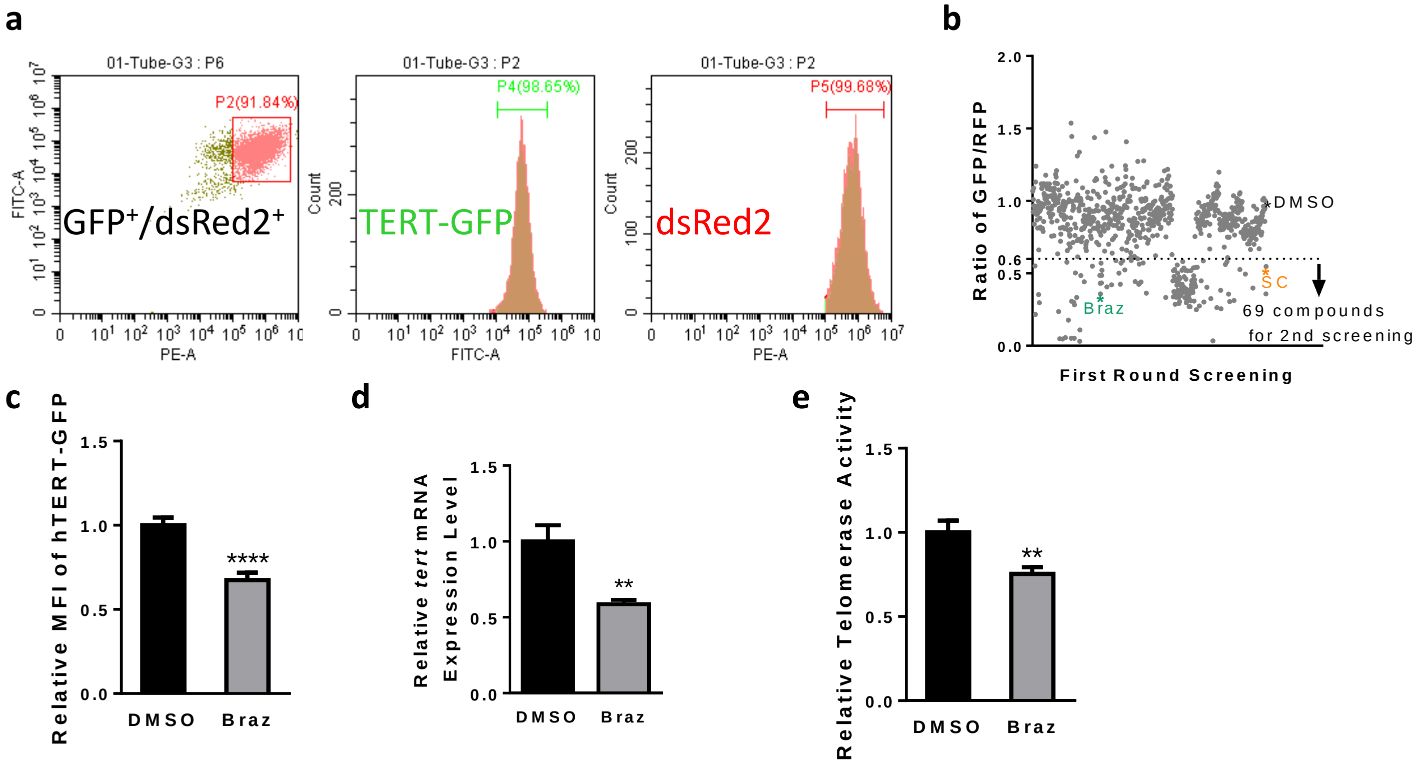

Figure S1 Compound screening for endogenous hTERT inhibitors. (a) Flow cytometry analyses of hTERT-P2A-GFP reporter, stable transfected dsRed2 served as an internal reference. (b) The first round screening of natural product pool. Compared to the value of GFP/dsRed2 in DMSO treating group, fold change of compound treating group over 40% has been listed for the second screening. (c) MFI quantification of GFP after 10 µM Braz treated for 48 hrs. (d) Quantitative real-time PCR assay of *TERT* mRNA in reporter cell line treated with 10 µM Braz for 24 hrs. (e) RQ-TRAP assay in reporter cell treated with 10 µM Braz for 48 hrs.

Table S1 The result of second screening.
Eight compounds have been identified for a reduction on GFP/RFP value. Data was presented as mean±SD, n=3.

**Compound Name**	**GFP**	**dsRed2**	**GFP/dsRed2**
Daunorubicin	0.4795±0.0136	3.8220±0.3747	0.1285±0.0127
Sanguinarium Chloride	0.4514±0.0285	1.0114±0.1226	0.4533±0.0615
Brazilin	0.6742±0.0360	1.4731±0.0553	0.4570±0.0074
Peruvoside	0.5644±0.0213	1.2459±0.1146	0.4588±0.0255
Celastrol	0.3772±0.0138	0.6338±0.0170	0.5496±0.0078
Dihydrocelastrol	0.3487±0.0115	0.6281±0.0091	0.5535±0.0240
Obtusaquinone	0.3858±0.0133	0.6596±0.0291	0.5848±0.0151
Methyl Gambogate Methyl Ether	0.3493±0.0059	0.5910±0.0192	0.5893±0.0126

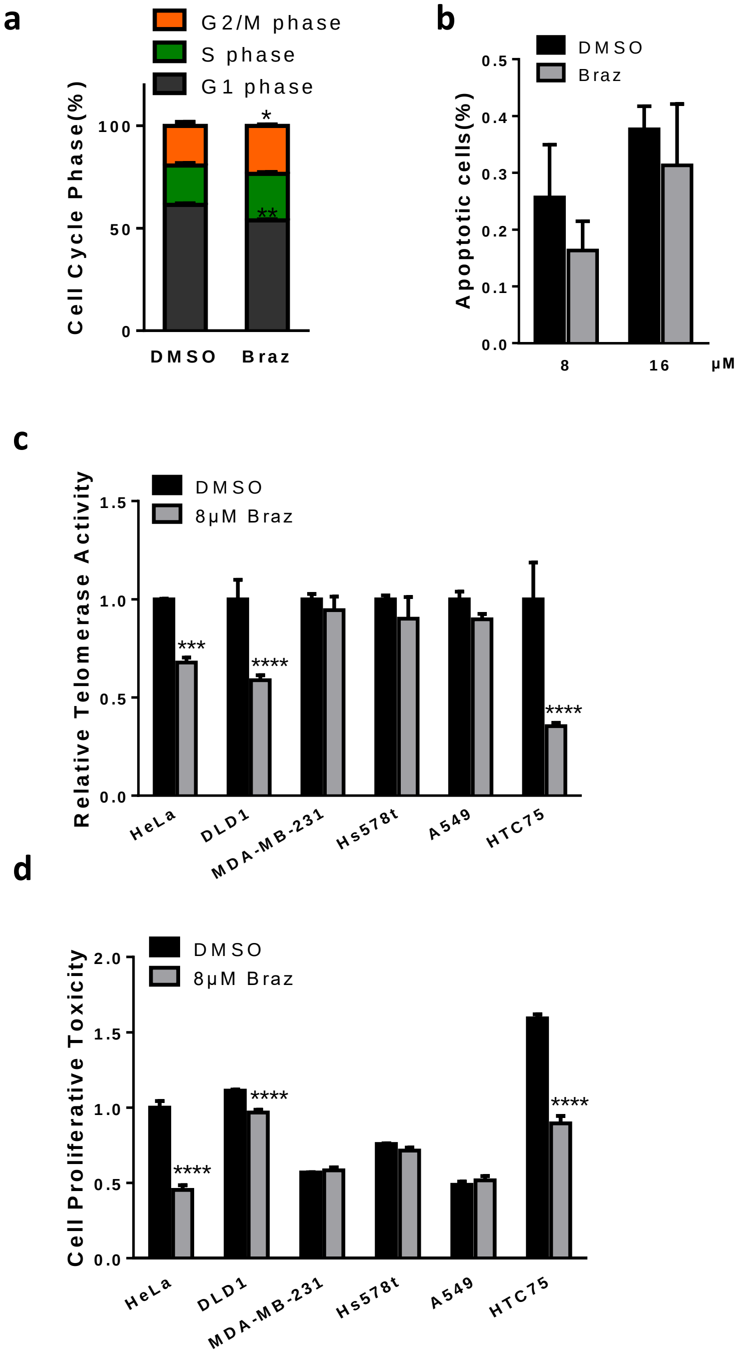

Figure S2 Effects of Braz treatment on cancer cells. (a) Quantification of cell cycle populations. (b) Quantification of apoptotic cells measured by Annexin-V and PI staining. (c) RTA level in different cancer cell lines following 8 μM Braz treatment. DMSO served as the control group. (d) CCK-8 assay showed cytotoxicity of Braz in different cancer cell lines.

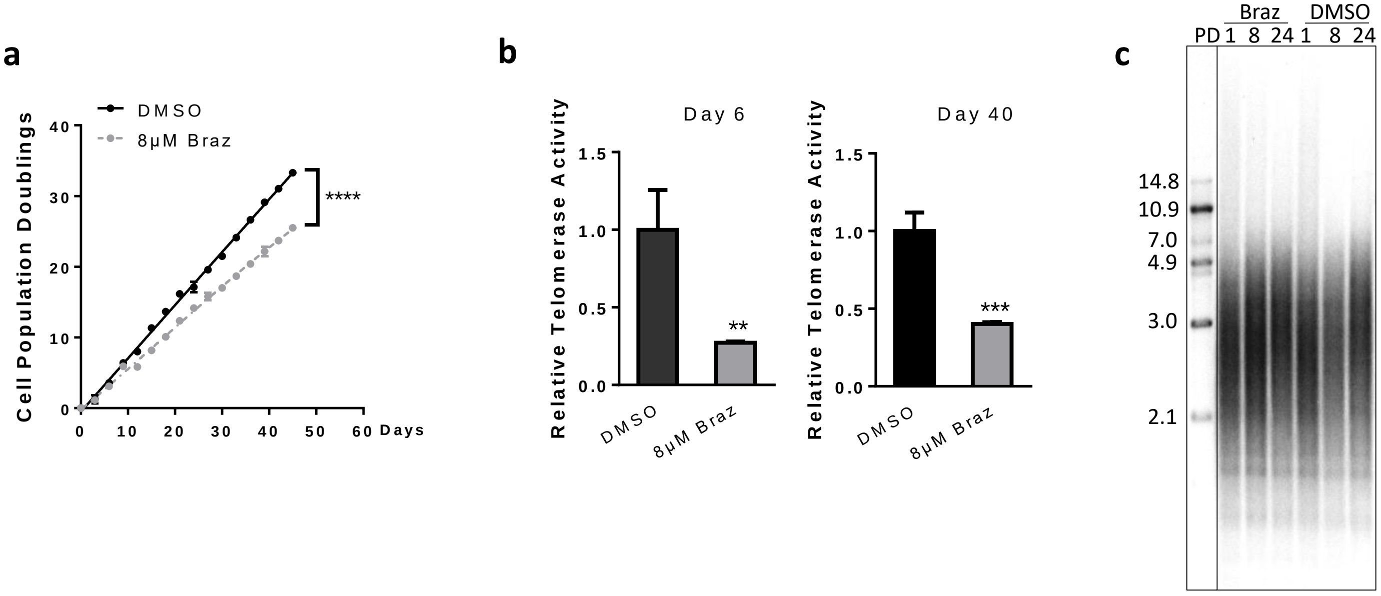

Figure S3 Analysis of HTC75 cell treated with Braz. (a) Cell growth curve of HTC75 cells under treatment of Braz. (b) Braz treated HTC75 cells were passaged over 40 days and detected for RTA by PCR-based TRAP. (c) Representative TRF assay showing telomere length after brazalin treatment.

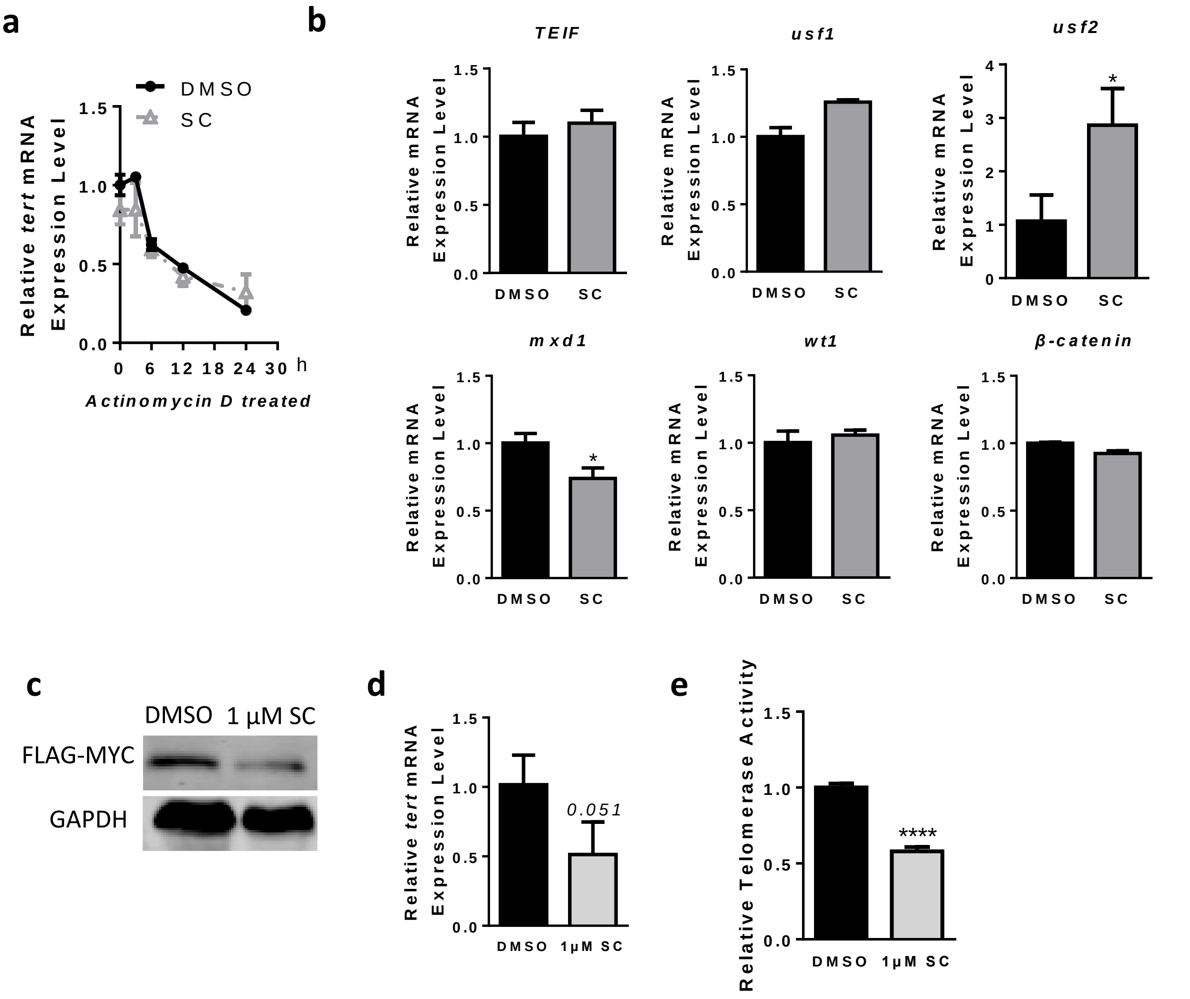

Figure S4 The mechanism of SC in regulating hTERT transcription.(a) TERT mRNA decay curves from SC treated HTC75 cells. Actinomycin D served as a transcription inhibitor. (b) The relative mRNA levels of other tested transcription factors which have been reported to regulate *TERT* transcription. (c)Western blot confirmed the overexpression of c-MYC in SC treated cancer cells. The mRNA level of *TERT* (d) and RTA (e) were detected when c-Myc was overexpressed.

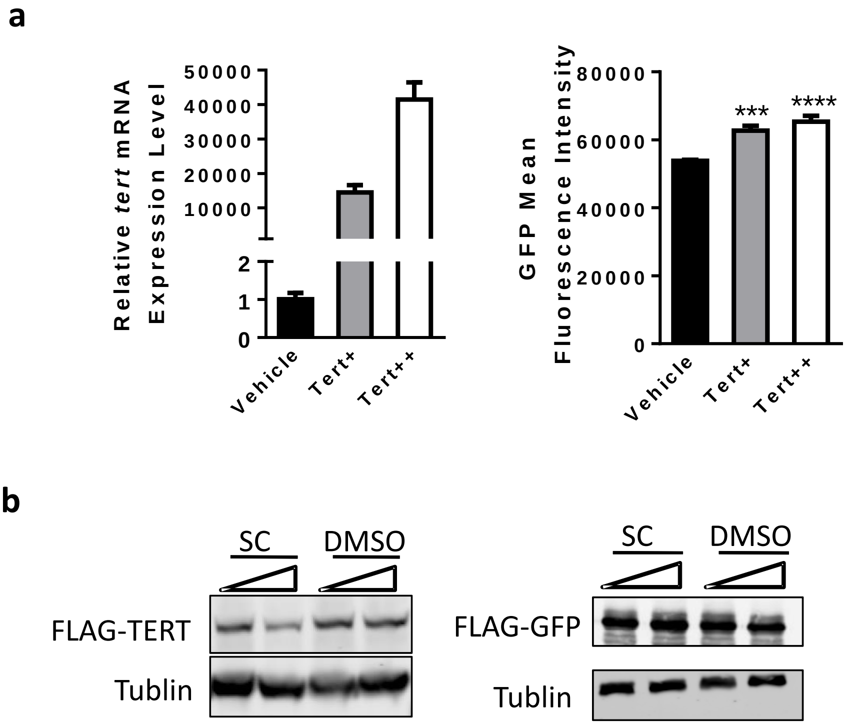

Figure S5 Discussion supplement. (a) The *TERT* transcriptional activity reporter contains a fragment of *TERT* core promoter and GFP as the indicative signal. The exogenous hTERT (FLAG-TERT) was overexpressed gradiently to the *TERT core promoter*-GFP reporter cell line (left). The endogenous hTERT transcriptional activity (indicated by *TERT core promoter*-GFP flurecence signal) was increased by overexpressing FLAG-hTERT protein (right). (b) SC repressed exogenous hTERT protein level (right), GFP served as the negative control (left).

Supplementary Methods

Real-time PCR

Total RNA was extracted using RNAiso Plus reagent according to the manufacturer's instructions (*TaKaRa*). First-stand cDNA was reverse transcribed with oligo d(T) using Maxima First Strand cDNA Synthesis Kit with dsDNase (*Thermo Fisher*). Quantitative real-time PCR was performed with 2×RealStar Green Power Mixture (with ROX) (*GeneStar*) on StepOnePlus™ Real-Time PCR system (*Applied Biosystems*). *Gapdh* was used as an internal control and data were calculated by comparative Ct method. Quantitative RT-PCR primers as follows: *hTERT* forward, 5'-TTCAAGTGCTGTCTGATTCCAAT-3' ; *hTERT* reverse, 5'-TCACGGAGACCACGTTTCAAA-3' ; *c-MYC* forward, 5'-GTCAAGAGGCGAACACACAAC-3' ; *c-MYC* reverse 5'-TTGGACGGACAGGATGTATGC-3'; *GAPDH* forward, 5'-GGAGCGAGATCCCTCCAAAAT-3'; *GAPDH* reverse, 5'-GGCTGTTGTCATACTTCTCATGG-3'; *p65* forward, 5'-AGGACATATGAGACCTTCAAGAGC-3'; *p65* reverse, 5'-CTCATCATAGTTGATGGTGCTCAG-3'; *MXD1* forward , 5'-TGAACATGGTTATGCCTCCA-3'; *MXD1* reverse, 5'-ACTTGATTCGGGTCCAAGTG-3'; *USF1* forward, 5'-CTGCTGTTGTTACTACCCAGG-3'; *USF1* reverse, 5'-TCTGACTTCGGGGAATAAGGG-3'; *USF2* forward, 5'-AGGGACCAGAAACAAGAGG-3'; *USF2* reverse, 5'-TAGTCCTCTCACCTGGAGGC-3'; *ETS* forward , 5'-CTGGGCATTCCAAAGAACCC-3'; *ETS* reverse, 5'-CCAGACTGAACTCATTGGTGG-3'; *TEIF* forward, 5'-TGACCCCGTTGGGAATATACC-3'; *TEIF* reverse, 5'-GAGGGCTTTCACGATCTGGTG-3'; *Sp1* forward, 5'-GGTTTACAAAGGAGGCTACAGA-3'; *Sp1* reverse, 5'-CCTCACCCCCCACTCTTAG-3'; *ß-catenin* forward, 5'-GCTTTCAGTTGAGCTGACCA-3'; *ß-catenin* reverse, 5'-CAAGTCCAAGATCAGCAGTCTC-3'; *WT1* forward, 5'-GCTATTCGCAATCAGGGTTACAG-3'; *WT1* reverse, 5'-TGGGATCCTCATGCTTGAATG-3';

Cell Proliferative inhibition

Adherent cell viability was determined using Cell Counting Kit-8 (CCK-8, *Dojindo*). 1000 cells in a volume of 100 μL were cultured in six

replicate wells in a 96-well plate and treated with serial dilutions of selected compounds for 48 hrs. The cells were then changed with 100 μL fresh complete medium contain 10% CCK-8 reagent and incubated at 37°C with 5% CO_2 for 2 hrs. Colorimetric assay was detected by Synergy HTX multi-reader (*BioTek*). Suspension cell viability was measured by CFSE (Carboxyfluorescein succinimidyl amino ester) staining. PBMCs were stained by CFSE for 20 min at 37°C in dark and then washed with PBS buffer twice. The CFSE labeled PBMCs were cultured with the compound or the according DMSO as control for 72 hrs and then analyzed by flow cytometry.

Cell cycle and Apoptosis

Cell cycle was analyzed by propidium iodide (PI) staining. After compound treatment for 48 hrs, cells were collected and fixed overnight in 70% ethanol at -20°C and then washed, pelleted and resuspended in 50 ng/μL PI, 100 μg/mL RNase A in PBS. Stained cells were measured by flow cytometry. Quantitative data was calculated by *ModFit LT* software. Apoptotic cells were examined by Annexin-V FITC antibody labeling and PI staining, and then measured by FACs (*Beckman* CytoFLEX S).

Cell Growth Curve

HTC75 cells were routinely cultured with the complete medium. Cells were reseeded into a 6-well plate with the initial number (Ni) of $1x10^5$. Cells were changed with fresh medium containing compound for 48 hours after adhesion to plate. The final number (Nf) of cells was counted by a hemocytometer and recorded. Population doubling was calculated using the equation: $PD=log_2$ (Nf-Ni).

Dual Luciferase Reporter Assay

The dual luciferase reporter assay was carried out as described (Zhou et al., 2013). Briefly, pGL3.0-hTERT promoter *Firefly* luciferase reporter and *Renilla* vector was transfected into cells. After 48 hrs cells were harvested, lysed and *Firefly*/*Renilla* luciferase activities were measured by using Dual Luciferase Reporter Assay Kit (Vazyme). The ratio of *Firefly* to *Renilla* activities indicated *hTERT* transcriptional activities.

Western Blotting and Immunofluorescence (IF)

Western blotting and IF were performed as previously described (Liu,

Safari, et al., 2004). The used antibodies are: mouse monoclonal anti-FLAG (*Sigma*), mouse monoclonal anti-GAPDH (*Protein tech*), mouse monoclonal anti-Tubulin (*Sigma*), mouse monoclonal anti-Human p53 (*Santa Crzu*), mouse monoclonal anti-Human p21 (*Calbiochem*), mouse monoclonal anti-Human p16 (*BD Pharmingen*), rabbit polyclonal anti-TRF2 (*Cell Signaling Technology*), rabbit monoclonal anti-TERT (abcam), rabbit monoclonal anti-p65 (*Cell Signaling Technology*), rabbit monoclonal anti-phospho-p65 (*Cell Signaling Technology*), Dylight 488 goat anti-rabbit IgG (*Invitrogen*), Alexa Flour® 555 Donkey Anti-Mouse IgG (*Invitrogen*), HRP anti-mouse (*Cell Signaling Technology*), HRP anti-rabbit (*Abcam*). Antibody BG4 tagged with His was purified by His-Ni column. Before the incubation of first antibody in IF, BG4 antibody was pre-incubated for 2 hrs at room temperature, and then the following steps were operated as usual.
